# Sulfotransferase 1C2 promotes hepatocellular carcinoma progression by enhancing glycolysis and fatty acid metabolism

**DOI:** 10.1002/cam4.5759

**Published:** 2023-03-07

**Authors:** Liya Jiang, Fang Xu, Chenglong Li, Ting Liu, Qianwei Zhao, Yixian Liu, Ying Zhao, Yamei Li, Zhendong Zhang, Xiaolei Tang, Jintao Zhang

**Affiliations:** ^1^ Henan Institute of Medical and Pharmaceutical Sciences Zhengzhou University Zhengzhou Henan China; ^2^ School of Life Sciences Zhengzhou University Zhengzhou Henan China; ^3^ School of Basic Medical Sciences Zhengzhou University Zhengzhou Henan China; ^4^ BGI College & Henan Institute of Medical and Pharmaceutical Sciences Zhengzhou University Zhengzhou Henan China; ^5^ Henan Key Laboratory of Tumor Epidemiology and State Key Laboratory of Esophageal Cancer Prevention & Treatment Zhengzhou University Zhengzhou Henan China; ^6^ Department of Veterinary Biomedical Sciences, College of Veterinary Medicine Long Island University Brookville New York USA; ^7^ Division of Regenerative Medicine, Department of Medicine, Department of Basic Science, School of Medicine Loma Linda University Loma Linda California USA

**Keywords:** fatty acid metabolism, glycolysis, hepatocellular carcinoma, HepG2, Huh7, sulfotransferase 1C2

## Abstract

**Background:**

Hepatocellular carcinoma (HCC) is aggressive liver cancer. Despite advanced imaging and other diagnostic measures, HCC in a significant portion of patients had reached the advanced stage at the first diagnosis. Unfortunately, there is no cure for advanced HCC. As a result, HCC is still a leading cause of cancer death, and there is a pressing need for new diagnostic markers and therapeutic targets.

**Methods:**

We investigated sulfotransferase 1C2 (SUTL1C2), which we recently showed was overexpressed in human HCC cancerous tissues. Specifically, we analyzed the effects of SULT1C2 knockdown on the growth, survival, migration, and invasiveness of two HCC cell lines, i.e., HepG2 and Huh7 cells. We also studied the transcriptomes and metabolomes in the two HCC cell lines before and after SULT1C2 knockdown. Based on the transcriptome and metabolome data, we further investigated the SULT1C2 knockdown‐mediated shared changes, i.e., glycolysis and fatty acid metabolism, in the two HCC cell lines. Finally, we performed rescue experiments to determine whether the inhibitory effects of SULT1C2 knockdown could be rescued via overexpression.

**Results:**

We showed that SULT1C2 overexpression promoted the growth, survival, migration, and invasiveness of HCC cells. In addition, SULT1C2 knockdown resulted in a wide range of gene expression and metabolome changes in HCC cells. Moreover, analysis of shared alterations showed that SULT1C2 knockdown significantly suppressed glycolysis and fatty acid metabolism, which could be rescued via SULT1C2 overexpression.

**Conclusions:**

Our data suggest that SULT1C2 is a potential diagnostic marker and therapeutic target for human HCC.

## Graphical abstract

Hepatocellular carcinoma is still a leading cause of cancer death, and there is a pressing need for new diagnostic markers and therapeutic targets. We showed that sulfotransferase 1C2 overexpression promoted hepatocellular carcinoma progression by enhancing glycolysis and fatty acid metabolism. Therefore, sulfotransferase 1C2 is a potential novel diagnostic and therapeutic target for this devastating disease.

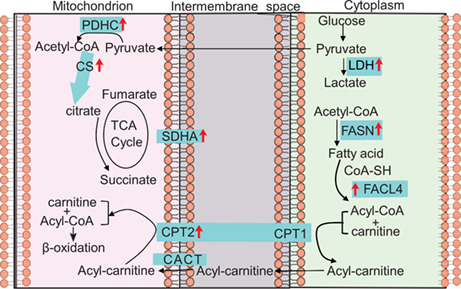

## 1 BACKGROUND

Liver cancer is a leading cause of cancer‐related death and one of the most prevalent cancers worldwide.^1^, ^2^ The majority of primary liver cancer is hepatocellular carcinoma (HCC). Available therapies have significantly prolonged survival time. In addition, numerous new targeted therapies and immunotherapies are under clinical trials to manage the disease.^3^, ^4^ Currently, except for those suitable for curative therapies, most HCCs gradually progress.^3^ Therefore, HCC treatment is still an unmet medical need. Consequently, it is imperative to understand HCC initiation and progression better to discover novel therapeutic targets.

Cancer development involves complex multi‐step processes caused by carcinogens. One of these processes is the accumulation of somatic genomic mutations.^4^, ^5^, ^6^ Somatic genomic alterations can cause cellular changes, such as activation of WNT‐β‐catenin pathway activation and fetal gene expressions.^7^, ^8^, ^9^, ^10^ In addition, the cellular changes can create a microenvironment that favors the outgrowth of the cells with driver mutations, leading to cancer progression.^11^, ^12^

Concerning somatic genomic mutations, sulfation is necessary for some carcinogens, such as heterocyclic amines (HCAs) produced during cooking, to produce DNA abducts via forming N‐C bonds at guanine bases, the underlying mechanisms of mutagenesis.^13^, ^14^, ^15^, ^16^, ^17^ Sulfation is mediated by sulfotransferase (SULTs). SULTs are phase II enzymes that transfer a negatively charged sulfate group (SO3‐) from the universal donor 3'‐phosphoadenosine 5′‐phosphosulfate (PAPS) to their substrates.^18^ Under physiological conditions, SULT substrates include endobiotics (e.g., hormones) and xenobiotics (e.g., drugs and chemicals). Sulfated endobiotics and xenobiotics become hydrophilic and easily excreted. Therefore, under physiological conditions, sulfation is a critical step of detoxification.^18^, ^19^

In addition to activating carcinogens, SULT‐mediated sulfation has been shown to regulate cancer progression. On the one hand, SULT‐mediated sulfation may suppress cancer progression. One example is SULT1E1. Its overexpression suppressed the growth of MCF‐7 cells (a breast adenocarcinoma cell line) in vitro and in vivo by arresting cell cycles and inducing apoptosis.^20^ It was suggested that the above anti‐cancer effect was due to estrogen inactivation via sulfation.^20^ Another example was that SULT2B1‐deficient mice had more gastric cancer incidence than wild‐type mice after the carcinogenic agent treatment.^21^ The cancer suppression effect of SULT2B1 was believed to be due to the removal of oxysterols via sulfation.^21^ On the other hand, SULT‐mediated sulfation can promote cancer progression. For example, the overexpression of carbohydrate sulfotransferase 11 (CHST11) in breast cancers was associated with poor survival.^22^ Although SULT1E1 overexpression suppressed MCF‐7 cell growth,^20^ the overexpression of CHST11 enhanced the proliferation, migration, and invasiveness of MCF‐7 cells.^22^

Based on the aforementioned previous findings, the role of SULTs in cancer development appears to be cell‐specific. In HCC, our recent studies showed that SULT1C2 was overexpressed in cancerous tissues compared to adjacent normal tissues in HCC patients.^23^ Additionally, a higher SULT1C2 expression was associated with a lower survival rate. Hence, our findings suggested that SULT1C2 played a role in HCC progression. However, currently identified SULT1C2 substrates did not explain the pro‐cancer effect, and the physiological substrates remain unknown.^24^ We reasoned that identifying SULT1C2 targets was essential for future detailed analysis of their physiological substrates. Hence, in this study, we hypothesized that SULT1C2 targeted key biological pathways necessary for HCC progression. To test our hypothesis, we performed RNA‐seq and metabolome analyses on human HCC cell lines before and after SULT1C2 knockdown to identify the targeted biological processes. We then further investigated the pathways that we believed were the primary targets of SULT1C2.

## 2 MATERIALS AND METHODS

### 2.1 Animals

Fourteen 3‐week‐old BALB/c nude mice were purchased from the Vital River Laboratories and housed in a standard barrier environment at Zhengzhou University animal facility. The animals were allowed for acclimation for about a week before experimentation. This study was approved by the Animal Care and Use Committee at Zhengzhou University (#2020‐30).

### 2.2 Human data

The RNA‐seq data (TPM format) of 374 HCC patients and their clinicopathological information were downloaded from TCGA public database (https://portal.gdc.cancer.gov). Spearman's rank coefficient was used to test correlations, and scatter plots were generated using the R package ggplot2(v.3.3.6).

### 2.3 Cell lines

Human hepatocellular carcinoma cell lines (HepG2, Huh7, SMMC7721, SNU449) and the normal LO2 hepatocytes were purchased from the Chinese Academy of Sciences. The LO2 and SNU449 cells were maintained in RPMI medium modified (HyClone) containing 10% FBS (Gibico). The HepG2, Huh7, and SMMC7721 cells were maintained in DMEM/high glucose medium (HyClone) containing 10% FBS (Gibico). All the cells were cultured at 37°C and 5% CO_2_.

### 2.4 Generation of stable SULT1C2 knockdown HepG2 and Huh7 cell lines

SULT1C2 expressions in HepG2 and Huh7 cells were knocked down using small hairpin RNA (shRNA). Briefly, shRNA targeting SULT1C2 and a negative control shRNA were purchased from Genechem Co., Ltd. The sequences of the shRNA are shown below:

Control‐shRNA: 5′‐TTCTCCGAACGTGTCACGT‐3′;

SULT1C2‐shRNA: 5′‐GCCCAGAATGAGAGGTTTGAT‐3′;

Twenty‐four hours before transduction, the cells (2 × 10^5^ cells/well) were added to a 6‐well plate and incubated at 37°C and 5% CO_2_. After reaching 70% confluence, the cells were transduced with the control‐shRNA (NC) or the SULT1C2‐shRNA (sh). Positively transduced cells were selected by puromycin (2 μg/mL). In addition, the constructs also expressed m‐Cherry (a red fluorescence) that was used for live imaging following in vivo injection of the parent and SULT1C2‐knockdown Huh7 cells (see “Mouse tumorigenesis experiment” in the following).

### 2.5 RNA isolation and RT‐qPCR

According to the manufacturer's instruction, total RNA was extracted from cells using TRIzol reagent (Invitrogen; Thermo Fisher Scientific, Inc). RNA (2 μg) was reverse‐transcribed into cDNA using the PrimeScript™ RT Reagent kit. qPCR was performed using SYBR Green (Roche Diagnostics). The thermocycling conditions were as follows: 95°C for 10 min; 35 cycles of 95°C for 60 s (denaturation), 56°C for 60 s (annealing), and 72°C for 2 min (extension); followed by 72°C for 6 min. Glyceraldehyde‐3‐phosphate dehydrogenase (GAPDH) served as an internal control. Relative gene expression was quantified using the 2^−ΔΔCT^ method.

The primer sequences of SULT1C2 are as follows:

Forward: 5′‐GGGAAGCCCTAAACCAAG‐3′;

Reverse: 5′‐GCATGAGACTGAACCCAAA‐3′;

The GAPDH primer sequences are as follows:

Forward: 5′‐CTCAAGGGCATCCTGGGCTA‐3′;

Reverse: 5′‐CGTCAAAGGTGGAGGAGTGG‐3′.

The SDHA (succinate dehydrogenase subunit A) primer sequences are as follows:

Forward: 5′‐GAGATGTGGTGTCTCGGTCCAT‐3′;

Reverse: 5′‐GCTGTCTCTGAAATGCCAGGCA‐3′.

The CS (citrate synthase) primer sequences are as follows:

Forward: 5′‐CACAGGGTATCAGCCGAACCAA‐3′;

Reverse: 5′‐CCAATACCGCTGCCTTCTCTGT‐3′.

The PDHC (pyruvate dehydrogenase) primer sequences are as follows:

Forward: 5′‐CAACTCCTGGACAACCCAATGC‐3′;

Reverse: 5′‐CTCCAAGGTCACAGTCAGCAGT‐3′.

The LDH (lactate dehydrogenase) primer sequences are as follows:

Forward: 5′‐TTGGAACTGGTGCCGTAGGCAT‐3′;

Reverse: 5′‐GACTGCCATGCTGAAGATCCATC‐3′.

The CPT2 (carnitine palmitoyltransferase 2) primer sequences are as follows:

Forward: 5′‐GCAGATGATGGTTGAGTGCTCC‐3′;

Reverse: 5′‐AGATGCCGCAGAGCAAACAAGTG‐3′.

The FASN (fatty acid synthase) primer sequences are as follows:

Forward: 5′‐TTCTACGGCTCCACGCTCTTCC‐3′;

Reverse: 5′‐GAAGAGTCTTCGTCAGCCAGGA‐3′.

The FACL4 (long‐chain‐fatty‐acid‐CoA ligase) primer sequences are as follows:

Forward: 5′‐GCTATCTCCTCAGACACACCGA‐3′;

Reverse: 5′‐AGGTGCTCCAACTCTGCCAGTA‐3′.

### 2.6 Western blotting

Cells were lysed using radioimmunoprecipitation assay (RIPA) buffer (Solarbio, Beijing, China) containing phenylmethylsulfonyl fluoride (PMSF; a serine protease inhibitor, Solarbio). Proteins were separated on a 10% SDS‐PAGE gel and transferred to a PVDF membrane. The membrane was blocked for 1.5 h in non‐fat powdered milk (BBI Life Sciences) and then incubated with a primary antibody at 4°C overnight. Primary antibodies used included anti‐SULT1C2 antibody (Abcam, ab243329), anti‐GAPDH antibody (Proteintech, 10494‐1‐AP), anti‐PDHC antibody (Cell Signaling Technology, 3205T), anti‐SDHA antibody (Cell Signaling Technology, 11998T), anti‐CS antibody (Proteintech, 16131‐1‐AP), anti‐LDH rabbit polyclonal antibody (Wanleibio, WL03271), anti‐recombinant CPT2 rabbit polyclonal antibody (BBI Life Sciences, D160201‐0010), anti‐FASN rabbit polyclonal antibody (BBI Life Sciences, D262701‐0010), anti‐FACL4 rabbit polyclonal antibody (ABGENT, AP2536B).

Following the primary antibody incubation, the membrane was washed three times with Tris‐buffered saline with Tween 20 (TBST) and incubated with a secondary antibody (HRP‐conjugated goat anti‐rabbit IgG, diluted in TBST) for 2 h. Subsequently, the membrane was washed three times with TBST. Protein bands were detected using the ECL chemiluminescence system.

### 2.7 Cell proliferation assay

Cell proliferation was determined using the Cell Counting Ki‐8 (CCK8, Dojindo). Briefly, 2000 cells/100 μL/well were seeded in a 96‐well plate. At 24, 48, 72, and 96 h, the cells were treated with the kit reagents for 2 h. Absorbance at 450 nm was then determined with a microplate reader (Bio‐Rad).

### 2.8 Colony‐forming assay

Four thousand cells/2 mL/well of HepG2 and Huh7 cells in DMEM culture medium were inoculated in a six‐well plate and cultured for 2 weeks. The cells were then washed with PBS, fixed with absolute methanol, and stained with 0.5% crystal violet. The stained colonies were photographed and manually calculated.

### 2.9 Mouse tumorigenesis experiments

Fourteen 4‐week‐old BALB/c nude mice were divided into two groups and marked with ear labels to distinguish them. 6 × 10^6^ Huh7 cells in 100 μL were inoculated subcutaneously under the right axilla of the vascular‐rich forelimb. When tumors were visible (typically 7 days after inoculation), Vernier caliper was used to measure their sizes every other day. Tumor volumes were calculated using the following formula: length × width^2^ × 0.5.

In addition, the mice were imaged in a small animal live imager 1 and 4 weeks after the cell injection. Immediately following the second imaging, the mice were euthanized. Tumors in the mice were isolated, weighed, and photographed. Subsequently, the tumor tissues were fixed in 4% paraformaldehyde for apoptosis detection using terminal deoxynucleotidyl transferase dUTP nick end labeling (TUNEL) assay (see “TUNEL assay” in the following).

### 2.10 Flow cytometry analysis of apoptosis

Apoptosis of HepG2 and Huh7 cells was analyzed using the Annexin V‐FITC reagent (KeyGEN: KGF001) by flow cytometry. Briefly, cells in the logarithmic phase were digested with trypsin without ethylenediaminetetraacetic acid (EDTA) and pelleted at 225 *g* for 4 min. The cells were washed twice with PBS. Then, 100 μL binding buffer (KGF005) was used to suspend the cells. Subsequently, 5 μL of the Annexin V‐FITC was added and mixed. The cells were incubated at room temperature in the dark for 15 min. The cells were then added with 400 μL binding buffer, mixed evenly, and analyzed on ACEA NOVOCYte3130 within 1 h.

### 2.11 TUNEL assay

Tumor tissues at week 4 were fixed in 4% paraformaldehyde, deparaffinized, and hydrated according to the conventional method of tissue section preparation (also see description in “Mouse tumorigenesis experiments”). The tissue sections were first incubated with the proteinase K working solution (Servicebio, G1205) at 37°C for 20 min and washed with phosphate‐buffered saline (PBS, PH 7.4) three times (5 min each time) afterward. Then, the tissue sections were incubated with the membrane‐breaking working solution (Servicebio, G1204) at room temperature for 20 min and washed with PBS (pH 7.4) three times (5 min each time). Subsequently, the tissue sections were incubated with the solution, which contained terminal deoxynucleotidyl transferase (TDT) and dUTP (Servicebio, G1501), at 37°C for 2 h. Finally, the tissue sections were counterstained with DAPI (Servicebio, G1012), mounted with the anti‐fluorescence quenching mounting tablets (Servicebio, G1401), and analyzed under a fluorescence microscope.

### 2.12 Detection of intracellular reactive oxygen species (ROS) production by H2DCFDA probe

This assay was performed using the KGAF018 kit (KeyGEN Bio Tech). Briefly, HepG2 or Huh7 cells were inoculated into a six‐well plate (6 × 10^5^ cells/well). After the cells were completely attached to the plate, the old medium was discarded, and the cells were washed twice gently with 1 mL PBS. Then, the cells were added with 1 mL/well of diluted working solution (1–10 μM working solution was prepared by diluting the 10 mM H2DCFDA original solution with PBS) and incubated at 37°C for 40 min. Subsequently, the working solution was discarded, and 2 mL DMEM/high glucose medium (HyClone) was added to each well. The cells were incubated at 37°C for 40 min. After the medium was discarded, the cells were washed gently with the DMEM/high glucose medium twice and examined under a fluorescence microscope. Pay attention to avoiding light during the experiment. Cells were counted under a microscope. Data were recorded as percentages of fluorescence^+^ areas from five random microscopic fields. We used “areas” but not “cells” because HepG2 cells tended to aggregate and therefore were difficult to count single cells.

### 2.13 Invasion and migration assay

For the invasion assay, matrigel (BD Biosciences) was melted overnight at 4°C. The melted matrigel was diluted in DMEM/high glucose medium (HyClone; matrigel: medium = 1:6). Then, 30 μL of the diluted matrigel was added into the upper chamber of a transwell plate with 8‐μm pore size (Corning). The plate was incubated at 37°C and 5% CO_2_ for 4 h (the matrigel solidified during this period). For the migration assay, there was no need to add matrigel into the upper chamber.

For both the invasion and migration assays, the DMEM/high glucose medium (HyClone) was added to the plate to moisten the membrane twice, 15 min each time. Then, 600 μL of the DMEM/high glucose medium containing 10% fetal bovine serum was added into the lower chambers. In addition, HepG2 (1 × 10^5^ cells/well) or Huh7 (5 × 10^4^ cells/well) cells in 600 μL of the DMEM/high glucose medium (without serum) were added into the up chambers. The plate was then incubated for 72 h for HepG2 cells and 48 h for Huh7 cells.

Following the incubation, cells that migrated into the membranes were counted. Briefly, the cells on the membrane surface were removed gently using cotton swabs. Subsequently, the membranes were fixed in absolute methanol and stained with 0.1% crystal violet solution. Cells in the membranes were counted under a microscope. Average cell numbers from five randomly chosen microscopic fields were presented.

### 2.14 Wound‐healing assay

Three parallel lines were marked in each well's center at the outside bottom of a 6‐well culture plate. Then, the transduced Huh7 cells (5 × 10^4^ cells/2 mL/well) were inoculated into the plate and cultured at 37°C and 5% CO_2_. After the cells reached 80% confluence, the medium was discarded. Wounds were drawn along the three parallel lines using a 200 μL pipette, and the scratched space was cleaned with PBS so that no residual cells were present. The plate was cultured at 37°C and 5% CO_2_. The cells were photographed at 0 and 48 h using a CKX53 microscope (Olympus), and migrated cells were counted. The percentage of migration was calculated as (0‐h scratched area − 48‐h scratched area) ÷ 0‐h scratched area.

### 2.15 RNA‐seq

Duplicate Huh7 and HepG2 cells with (sh) and without (NC) SULT1C2 knockdown (5 × 10^6^ cells/sample) were submitted to Novogene Bioinformatics Technology Co. Ltd for transcriptome sequencing. Briefly, RNA was extracted from the cells, and mRNA with polyA tail by Oligo (dT) was enriched by magnetic beads. Sequencing was performed on the Illumina sequencing platform. The data were analyzed by DESeq2 for differentially expressed genes.^25^ The clusterProfile software was used to analyze the KEGG pathway enrichment. padj (*p*‐value adjusted) or *p* < 0.05, i.e., −log10(padj or *p*) > 1.301, was considered significant.

### 2.16 Measurement of oxygen consumption rate

The oxygen consumption rate of HepG2 and Huh7 cells was measured using the MitoXpress‐Xtra kit (Cat#: MX‐100/5; Lucxel Biosciences Ltd.) on the CLARIOstar ACU instrument (BMG LABTECH). The main component of this kit is oxygen‐sensitive fluorescein probe solution and mineral oil. Briefly, HepG2 and Huh7 cells (60,000 cells/100 μL/well) with (sh) and without (NC) SULT1C2 knockdown were seeded in a 96‐well plate and cultured at 37°C and 5% CO_2_. The following day, the culture plate's old medium was discarded and replaced with 150 μL/well of preheated fresh medium. Then, 10 μL/well of the probe solution was added to each well, and the plate was sealed with two drops of preheated mineral oil. The plate was then placed in the prewarmed CLARIOstar ACU instrument, and the oxygen consumption rates in the cells were measured for 100 min.

### 2.17 Measurement of extracellular acidification rates

The extracellular acidification rate of HepG2 and Huh7 cells was measured using the pH‐extra kit (Cat#: pH −100; Lucxel Biosciences Ltd.) on the CLARIOstar ACU instrument (BMG LABTECH). The main component of this kit is the pH‐sensitive fluorescein probe. Briefly, HepG2 and Huh7 cells (60,000 cells/100 μL/well) with (sh) and without (NC) SULT1C2 knockdown were seeded in a 96‐well plate and cultured at 37°C and 5% CO_2_. The next day, the culture plate's old medium was discarded, and the cells were washed with the respiration buffer twice (100 μL/well each time). The plate was then added with 150 μL/well of the respiration buffer and 10 μL/well of the probe solution. If the total extracellular acidification rate (T‐ECAR) was measured, the plate was sealed with two drops of preheated mineral oil. However, the plate was not sealed if the lactic acid extracellular acidification rate (L‐ECAR) was examined. The plate was then placed in the prewarmed CLARIO Star‐ACU instrument, and the acidification rate was determined for 100–250 min.

### 2.18 Metabolomics analysis

Five replicates of Huh7 cells with (sh) and without (NC) SULT1C2 knockdown (approximately 1 × 10^7^ cells/sample) were collected. The cells were washed twice with ice‐cold PBS and once with ice‐cold saline. Then, the cells were reconstituted with 1 mL of pre‐cooled methanol/acetonitrile/water solution (2:2:1, v/v/v) and transferred into 1.5 mL Eppendorf tubes, and stored at −80°C. The samples were further processed and analyzed by Applied Protein Technology Co., Ltd. using ultra‐high‐pressure liquid chromatography with a hydrophilic interaction liquid chromatography (HILIC) column.

The raw MS data (wiff.scan files) were converted to MzXML files using ProteoWizard MSConvert before being imported into freely available XCMS software for analysis.^26^ For peak picking, the following parameters were used: centWave *m*/*z* = 25 ppm, peakwidth = c (10, 60), prefilter = c (10, 100). For peak grouping, bw = 5, mzwid = 0.025, minfrac = 0.5 were used. CAMERA (Collection of Algorithms of MEtabolite pRofile Annotation) was sued for annotation of isotopes and adducts. In the extracted ion features, only the variables having more than 50% of the nonzero measurement values in at least one group were kept. Compound identification of metabolites was performed by comparing the m/z value (<25 ppm) and MS/MS spectra with an in‐house database established with available authentic standards. The identified compounds were then analyzed for differential expression between groups using univariate statistical analysis. KEGG pathway analysis was performed using the Kyoto Encyclopedia of Genes and Genomes database (http://www.genome.jp/kegg/).

### 2.19 Generation of stable SULT1C2‐overexpressing HepG2 and Huh7 cell lines

Lentivirus expressing the SULT1C2 gene (LV‐SULT1C2) was purchased from Genechem Co., Ltd. The primer sequences used to obtain the SULT1C2 gene are as follows:

Reverse: CACACATTCCACAGGAATTTCAGAGTTCCATGCAGAAGTTTATG;

Forward: CCAACTTTGTGCCAACCGGTCGCCACCATGGCCCTGACCTCAGACCTG.

The LV‐SULT1C2 virus was then used to overexpress SULT1C2 in HepG2 and Huh7 cells. Briefly, 24 h before transduction, the cells (2 × 10^5^ cells/well) were added to a 6‐well plate and incubated at 37°C and 5% CO_2_. After reaching 70% confluence, the cells were transduced with the LV‐SULT1C2. The calculation formula for the volume of the added virus was: *V* = (MOI value × number of cells)/virus titer, where the virus titer was 1 E+9 TU/mL, the MOI of Huh7 cells was 5, and that of HepG2 cells was 10. Finally, the positively transduced cells were selected by puromycin (2 μg/mL).

### 2.20 Statistical analyses

Independent student's *t*‐test or ANOVA was performed using GraphPad Prism 7.0. Data were presented as mean ± SD (standard deviation). Three repeats were included in the cumulative data. Differences were considered significant at *p* < 0.05.

## 3 RESULTS

### 3.1 SULT1C2 expression correlates with other potential cancer drivers in human HCCs

We recently found that SULT1C2 was overexpressed in cancerous tissues compared to adjacent normal tissues in three HCC patients (*p* < 0.001).^23^ In addition, TCGA database analysis also showed that, compared to adjacent normal tissues, 66% of HCC cancerous tissues displayed significantly higher expression levels of SULT1C2 (1.337 ± 1.392 vs. 0.307 ± 0.364, *p* < 0.001). Furthermore, a higher SULT1C2 expression was associated with a lower survival rate.^23^ Hence, our data suggest that SULT1C2 can be a potential HCC progression driver.

**FIGURE 1:**
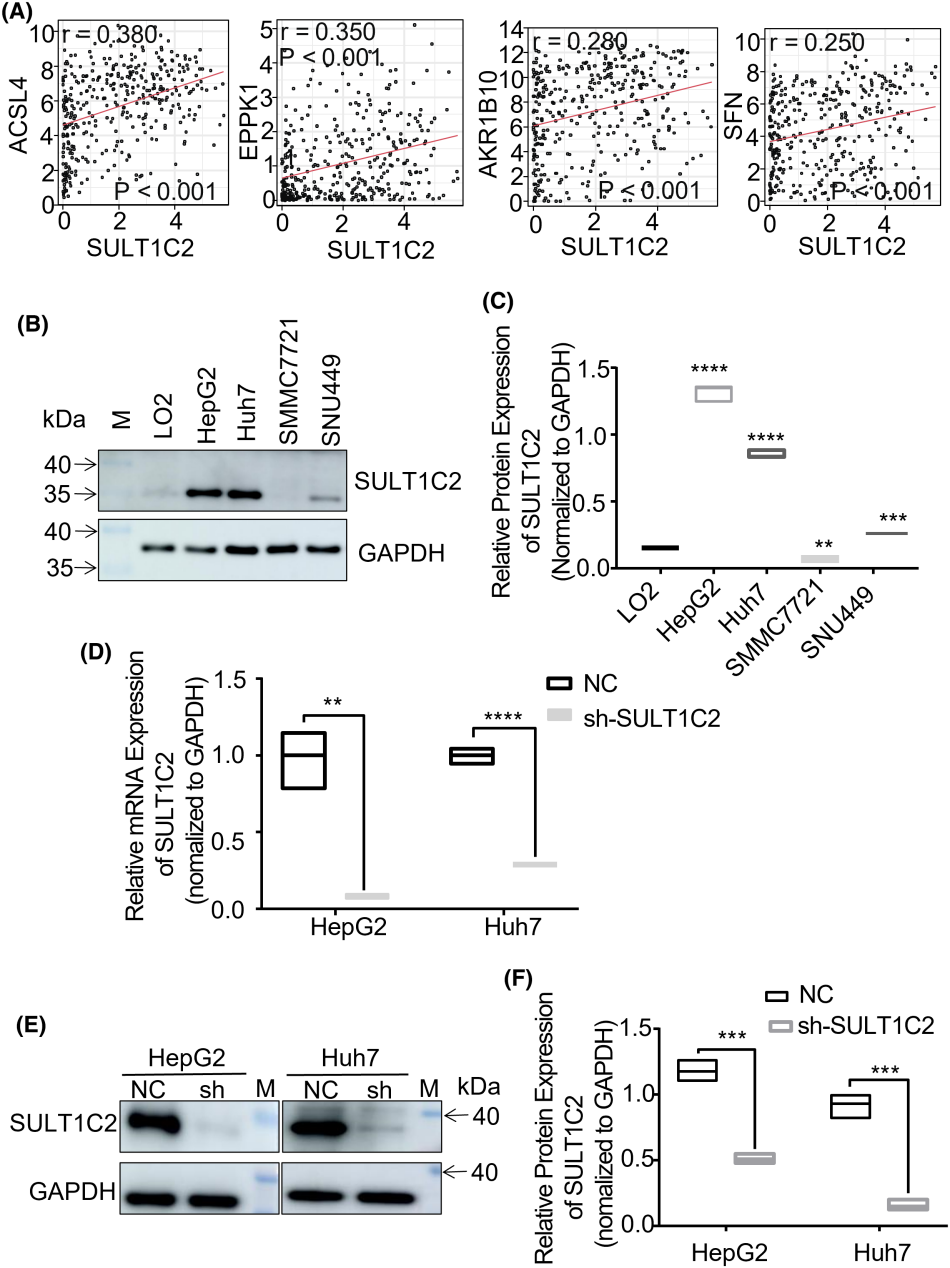
SULT1C2 expression in human HCC correlated with other potential HCC drivers. (A) Data show the correlation of SULT1C2 mRNA expression with ACSL4 (FACL4), EPPK1, AKR1B10, and SFN mRNA expression among 374 HCC patients, as analyzed by Spearman correlation. (B) SULT1C2 protein expressions in the normal human liver cell line (L02) and the human HCC cell lines (HepG2, Huh7, SMMC7721, and SNU449) were examined by Western Blot. Data show representative images. “M” represents the visual protein Marker. (C) Quantitative data show SULT1C2 protein expressions in L02, HepG2, Huh7, SMMC7721, and SNU449. (D) SULT1C2 expressions in HepG2 and Huh7 cells were knocked down using shRNA as described in Section 2. Data show SULT1C2 mRNA expressions in HepG2 and Huh7 cells with (sh) and without (NC) SULT1C2 knockdown. (E) Representative images of Western Blot show SULT1C2 protein expressions in HepG2 and Huh7 cells with (sh) and without (NC) SULT1C2 knockdown. “M” represents the visual protein Marker. (F) Quantitative data show SULT1C2 protein expressions in HepG2 and Huh7 cells with (sh) and without (NC) SULT1C2 knockdown. Where applicable, **p < 0.01, ***p < 0.001, ****p < 0.0001. ANOVA test.

In this regard, several novel molecules, e.g., Acyl‐CoA synthetase long‐chain family member 4 (ACSL4), EPPK1, aldo‐keto reductase family 1 member B10 (AKR1B10), and stratifin (SFN), were recently shown being overexpressed in human HCC tissues.^27^, ^28^, ^29^, ^30^, ^31^, ^32^, ^33^ For this reason, we analyzed the relationship between SULT1C2 and other overexpressed molecules recently discovered using The Cancer Genome Atlas (TCGA) database.^34^ Our data showed that the expression of SULT1C2 in HCC cancerous tissues positively correlated with the recently reported overexpressed molecules (Figure 1A).

### 3.2 SULT1C2 knockdown inhibits HCC cell growth ex vivo and in vivo

The above findings prompted us to understand how the SULT1C2 overexpression might affect HCC progression. To address this question, we first analyzed SULT1C2 expression in standard HCC cell lines, including LO2, HepG2, Huh7, SMMC7721, and SNU449. LO2 is derived from normal hepatocytes, while HepG2, Huh7, SMMC7721, and SNU449 are hepatocellular carcinoma cells. Our data showed that HepG2, Huh7, and SNU449 but not SMMC7721, compared to LO2, expressed significantly higher levels of SULT1C2 (Figure 1B,C). The data suggested that most but not all HCC cells expressed high levels of SULT1C2, which was consistent with the findings in human HCC cancerous tissues (Figure 1A).^23^

To determine the role of high‐level SULT1C2 expression in HCC progression, we decided to investigate further how the high‐level SULT1C2 expression impacted the biological functions of HepG2 and Huh7 cells because they expressed the highest SULT1C2 levels among the four cell lines examined. Our approach was to identify the biological processes in HepG2 and Huh7 cells that were altered after SULT1C2 knockdown (Figure 1D–F).

**FIGURE 2:**
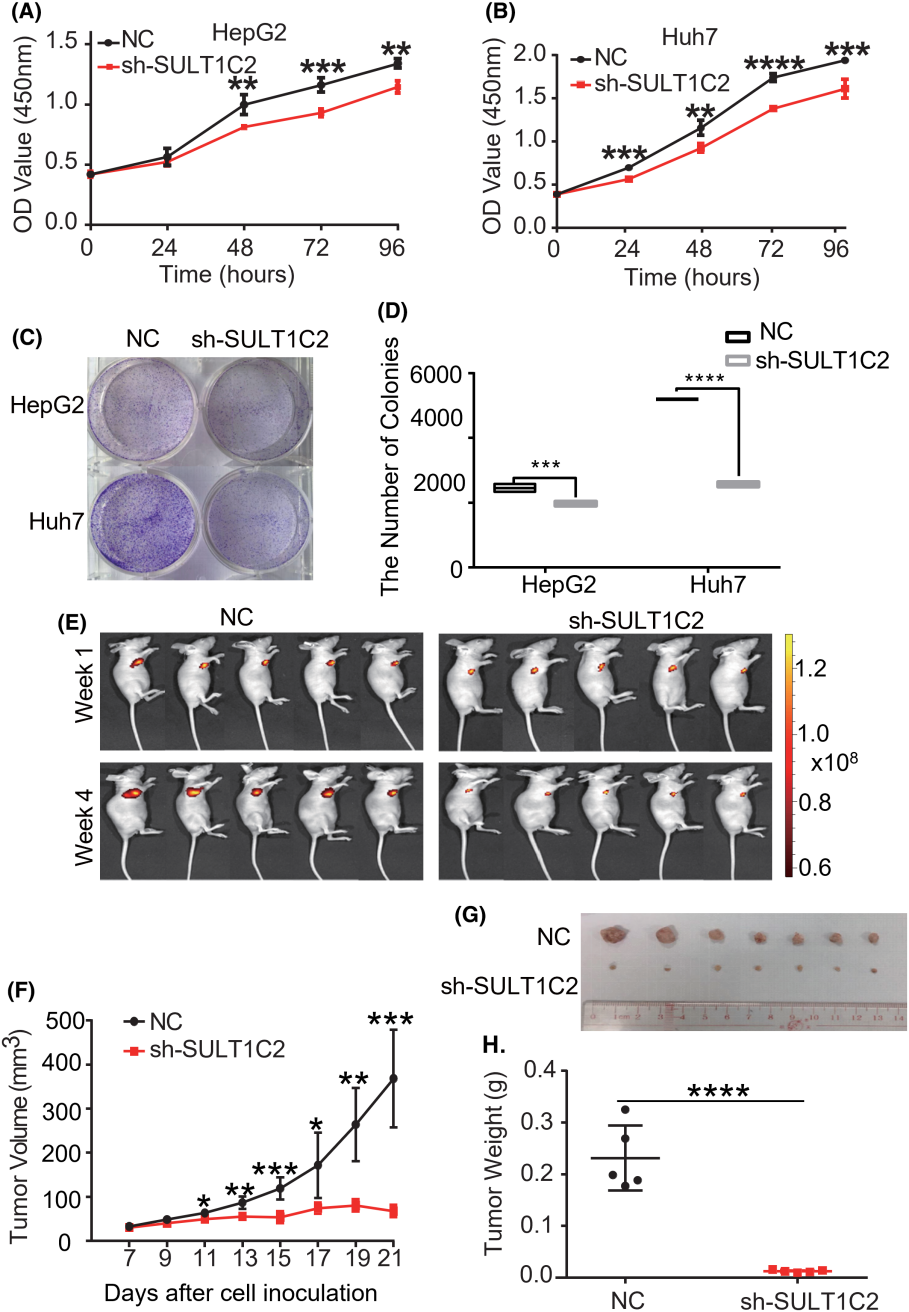
SULT1C2 knockdown inhibited HCC cell growth ex vivo and in vivo. (A, B) The proliferation of control (NC) and SULT1C2 knockdown (sh) HepG2 (A) and Huh7 (B) cells were measured using the CCK‐8 assay described in Section 2. Data show OD values at 0, 24, 48, 72, and 96 h after cell culture initiation. (C) Colony‐forming ability of control (NC) and SULT1C2 knockdown (sh) HepG2 and Huh7 cells was examined as described in Section 2. Data show representative images of colony formation. (D) Cumulative data show colonies formed by control (NC) and SULT1C2 knockdown (sh) HepG2 and Huh7 cells. (E) 6 × 10^6^ control (NC) and SULT1C2 knockdown (sh) Huh7 cells were subcutaneously inoculated into 4‐week‐old BALB/c nude mice. At weeks 1 and 4, the mice were imaged in a small animal live imager. (F) Tumor size was measured at the indicated days in the BALB/c nude mice that were inoculated with control (NC) and SULT1C2 knockdown (sh) Huh7 cells. (G) Images of the tumors isolated from the BALB/c nude mice that were inoculated with control (NC) or SULT1C2 knockdown (sh) Huh7 cells for 4 weeks. (H) Data show weights of the tumors isolated from the BALB/c nude mice that were inoculated with control (NC) or SULT1C2 knockdown (sh) Huh7 cells for 4 weeks. Where applicable, *p < 0.05, **p < 0.01, ***p < 0.001, ****p < 0.0001. ANOVA test.

Firstly, we analyzed the effects of SULT1C2 knockdown on HepG2 and Huh7 cell growth ex vivo and in vivo. First, our ex vivo data showed that SULT1C2 knockdown significantly inhibited the proliferation (Figure 2A,B) and colony formation (Figure 2C,D) of HepG2 and Huh7 cells ex vivo. In vivo, we subcutaneously inoculated control (NC) and SULT1C2 knockdown (sh) Huh7 cells into 4‐week‐old BALB/c nude mice. At weeks 1 and 4, the mice were imaged in a small animal live imager. Our data showed that SULT1C2 knockdown Huh7 cells' tumor sizes were significantly smaller than the controls (Figure 2E–G). In addition, the tumors were isolated and weighed at week 4, revealing that the weights of SULT1C2 knockdown Huh7 cells were significantly lower compared to the controls (Figure 2H). Hence, our data demonstrate that SULT1C2 knockdown substantially reduces the HCC growth ex vivo and in vivo.

### 3.3 SULT1C2 knockdown promotes the apoptosis of HCC cells

**FIGURE 3:**
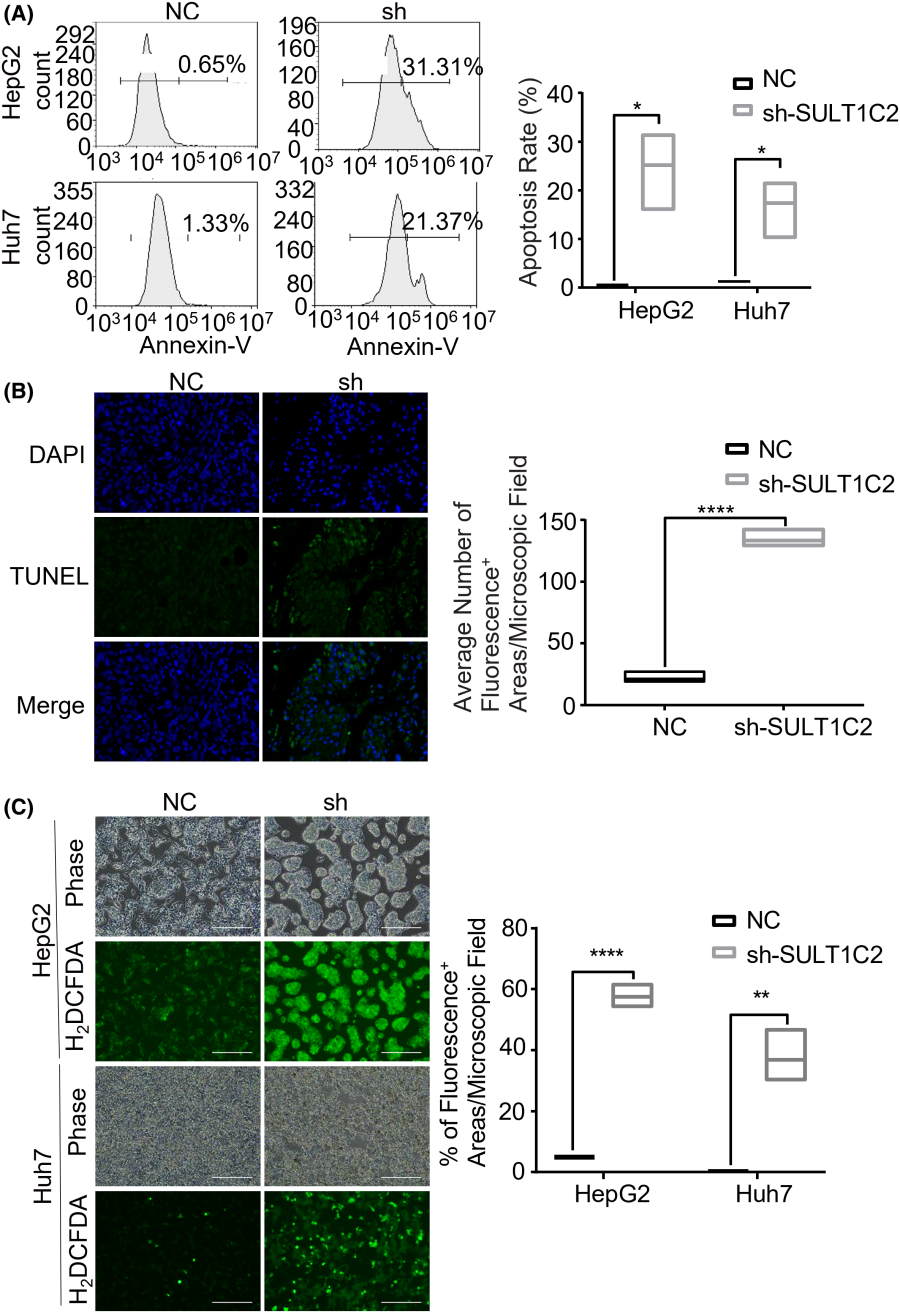
SULT1C2 knockdown promoted the apoptosis of HCC cells. (A) Control (NC) and SULT1C2 knockdown (sh) HepG2 and Huh7 cells were stained with Annexin‐V and analyzed by fluorescence‐activated cell sorting (FACS). Representative FACS plots (left panel) and cumulative data (right panel) are shown. (B) Tumor tissues from the BALB/c nude mice that were inoculated with control (NC) and SULT1C2 knockdown (sh) Huh7 cells were analyzed for apoptosis by terminal deoxynucleotidyl transferase dUTP nick end labeling (TUNEL) assay. Representative images (left panels, apoptotic cells are stained with FITC [green fluorescence]) and cumulative data (right panel) are shown. Scale bar is 100 μm. (C) Intracellular reactive oxygen species (ROS, green fluorescence) in control (NC) and SULT1C2 knockdown (sh) HepG2 and Huh7 cells were determined as described in Section 2. Representative images (left panel) and cumulative data (right panel) are shown. Where applicable, *p < 0.05. ***p < 0.001, ****p < 0.0001. Independent‐Sample *t*‐test. The scale bar is 200 μm.

Next, we examined the effects of SULT1C2 knockdown on HepG2 and Huh7 cell apoptosis. When analyzed by fluorescence‐activated cell sorting (FACS), HepG2 and Huh7 cells contained significantly increased numbers of Annex‐V^+^ cells after SULT1C2 knockdown, suggesting elevated apoptosis (Figure 3A).

To further determine the effects of SULT1C2 knockdown on HCC apoptosis, we used TUNEL assay to analyze the apoptosis of Huh7‐inoculated tumor tissues (Figure 2G). Consistent with the in vitro finding (Figure 3A), our data showed that SULT1C2 knockdown led to increased apoptosis in the Huh7‐inoculated tumor tissues (Figure 3B).

Concerning cancer cell apoptosis, high levels of reactive oxygen species (ROS) trigger apoptosis.^35^ It is worth mentioning that cancer cells, compared to normal cells, usually contain higher ROS levels, and a moderate increase in ROS levels is beneficial to cancer cells.^35^, ^36^ For this reason, we examined the effects of SULT1C2 knockdown on the ROS levels in HepG2 and Huh7 cells. Our data showed that SULT1C2 knockdown significantly increased ROS levels in HepG2 and Huh7 cells (Figure 3C), suggesting that SULT1C2 prevents the overproduction of ROS in HCC cells, consistent with the above apoptosis analyses (Figure 3A,B).

### 3.4 SULT1C2 knockdown decreases the migration and invasiveness of HCC cells

**FIGURE 4:**
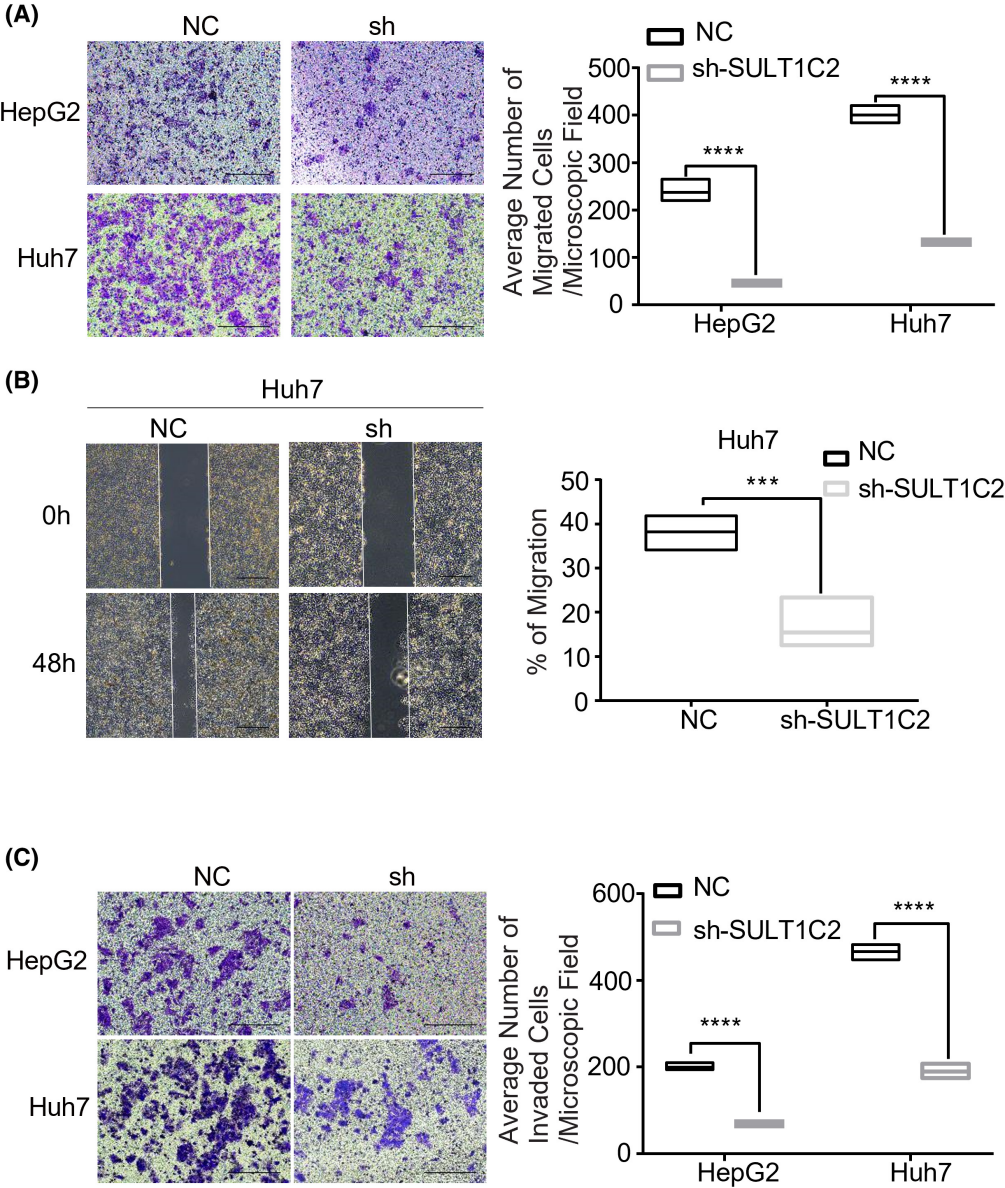
SULT1C2 knockdown decreased the migration and invasiveness of HCC cells. (A) The migration of control (NC) and SULT1C2 knockdown (sh) HepG2 and Huh7 cells were examined as described in Section 2. Representative images (left panels) and cumulative data (right panels) are shown. The scale bar is 200 μm. (B) Wound healing assay was performed on control (NC) and SULT1C2 knockdown (sh) Huh7 cells, as described in Section 2. The scale bar is 500 μm. (C) The invasiveness of control (NC) and SULT1C2 knockdown (sh) HepG2 and Huh7 cells were examined as described in Section 2. Representative images (left panels) and cumulative data (right panels) are shown. ***p < 0.001, ****p < 0.0001. Independent‐Sample *t*‐test. The scale bar is 200 μm.

We also investigated the migration and invasiveness of HepG2 and Huh7 cells. Transwell migration assay showed that SULT1C2 knockdown significantly decreased the migratory ability of HepG2 and Huh7 cells (Figure 4A), which was further supported by the wound healing assay (Figure 4B). In addition, the transwell invasiveness assay demonstrated that SULT1C2 knockdown significantly reduced the invasive ability of HepG2 and Huh7 cells (Figure 4C).

Collectively, the data from analyzing the growth, apoptosis, migration, and invasiveness of HepG2 and Huh7 cells support that SULT1C2 promotes HCC progression.

### 3.5 SULT1C2 knockdown results in a wide range of changes in gene expression and metabolome in HCC cells

The endogenous substrates of SULT1C2 remain unknown.^19^ The well‐characterized SULT1C2 substrates are phenols from exogenous compounds,^37^ which, however, do not explain the effects of SULT1C2 knockdown in HCC cells. For this reason, we decided to compare the transcriptomes in HepG2 and Huh7 cells before and after SULT1C2 knockdown. Our transcriptome analysis showed that SULT1C2 knockdown led to a wide range of changes in gene expression in HepG2 and Huh7 cells (Figure S1A–C). We then performed Kyoto Encyclopedia of Genes and Genomes (KEGG) pathway analysis on the changed gene expression. Our data demonstrated that the altered genes were enriched in multiple pathways (Figure S1D,E). In addition, the pathways affected by SULT1C2 knockdown were vastly different between HepG2 and Huh7 cells. However, it was evident that glycolysis/gluconeogenesis and fatty acid metabolism were altered in both HepG2 and Huh7 cells.

The above data suggest that one crucial SULT1C2 function is to regulate metabolism in cancer cells. This novel finding is important because metabolic reprogramming has been shown essential for cancer progression.^38^, ^39^ Hence, we further analyzed the metabolome in Huh7 cells before and after SULT1C2 knockdown. Consistent with the transcriptome analysis, metabolome analysis showed a wide range of changes in metabolites in Huh7 cells after SULT1C2 knockdown (Figure S2A). Another consistent finding was that quantities of the metabolites associated with glucose and fatty‐acid metabolisms were significantly reduced after SULT1C2 knockdown (Figure S2B,C).

Because glucose and fatty acid metabolisms are closely associated with energy generation and essential for cancer cell growth, we examined the metabolites related to energy generation. Our data showed that the changed metabolites after SULT1C2 knockdown were also enriched in the pathways involved in energy generation (Figure S2D).

### 3.6 SULT1C2 knockdown significantly suppresses the expression of the proteins necessary for glycolysis and fatty acid metabolism in HepG2 and Huh7 cells

**FIGURE 5:**
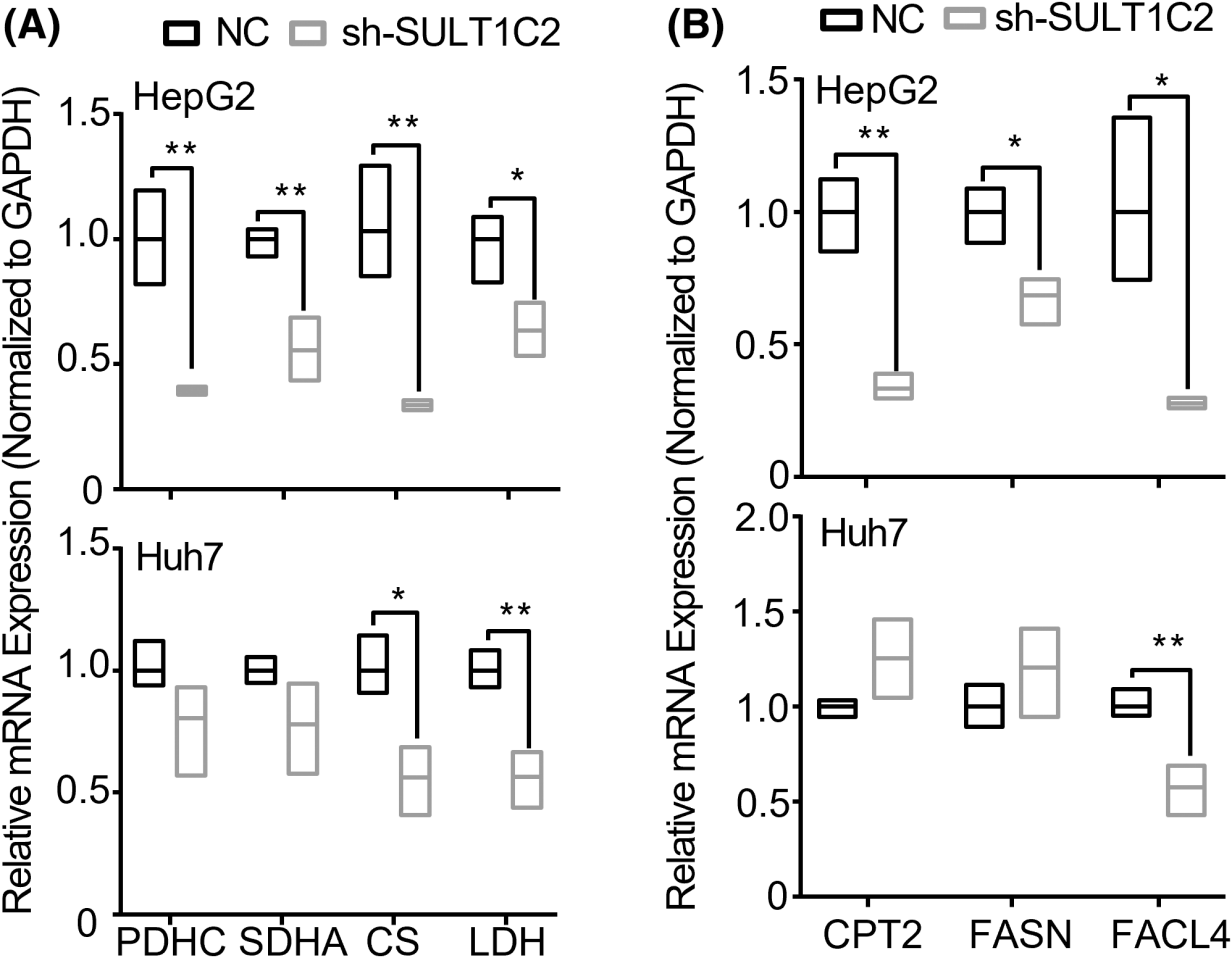
Effects of SULT1C2 knockdown on the mRNA expressions of selected genes necessary for glycolysis and fatty acid metabolism in HepG2 and Huh7 cells. (A) The mRNA expressions of PDHC, SDHA, CS, and LDH in HepG2 (up panel) and Huh7 (lower panel) cells with (sh) and without (NC) SULT1C2 knockdown. (B) The mRNA expressions of CPT2, FASN, and FACL4 in HepG2 (up panel) and Huh7 (lower panel) cells with (sh) and without (NC) SULT1C2 knockdown. *p < 0.05, **p < 0.01. Independent‐Sample *t*‐test.

**FIGURE 6:**
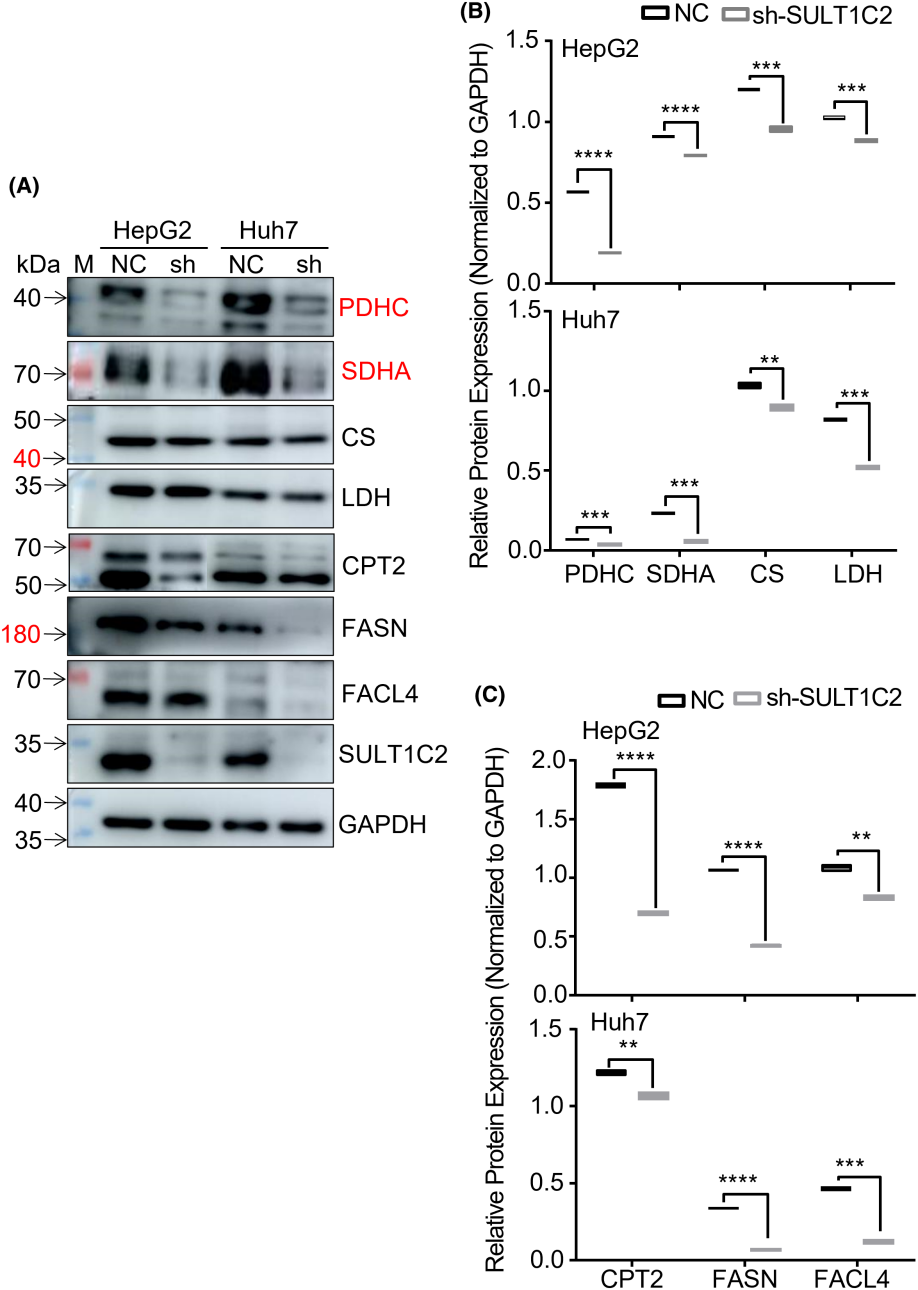
SULT1C2 knockdown significantly suppressed the expressions of the proteins necessary for glycolysis and fatty acid metabolism in HepG2 and Huh7 cells. (A) Representative western blot images showing the expressions of the proteins related to oxidative phosphorylation (PDHC, SDHA, CS, and LDH) and fatty acid metabolism (CPT2, FASN, and FACL4) in HepG2 and Huh7 cells with (sh) and without (NC) SULT1C2 knockdown. “M” represents the visual protein Marker. (B) Quantitative data showing the expressions of the proteins necessary for oxidative phosphorylation (PDHC, SDHA, CS, and LDH) in HepG2 (upper panel) and Huh7 (lower panel) cells with (sh) and without (NC) SULT1C2 knockdown. (C) Quantitative data showing the expressions of the proteins required for fatty acid metabolism (CPT2, FASN, and FACL4) in HepG2 (upper panel) and Huh7 (lower panel) cells with (sh) and without (NC) SULT1C2 knockdown. Where applicable, **p < 0.01, ***p < 0.001, ****p < 0.0001. Independent sample *t*‐test.

Based on the transcriptome and metabolome analyses, we examined the effects of SULT1C2 knockdown on the expression of glycolysis and fatty acid metabolism enzymes in HepG2 and Huh7 cells. Specifically, we analyzed lactate dehydrogenase (LDH) that is associated with anaerobic glycolysis; pyruvate dehydrogenase (PDHC), citrate synthase (CS), and succinate dehydrogenase subunit A (SDHA) that are related to oxidative phosphorylation; fatty acid synthase (FASN), fatty acid coenzyme ligase 4 (FACL4), and carnitine palmitoyltransferase 2 (CPT2) that are required for fatty acid metabolism. When mRNAs were analyzed using RT‐qPCR, our data showed that SULT1C2 knockdown significantly decreased the gene expression of the above enzymes in HepG2 cells (Figure 5A,B, upper panels). However, in Huh 7 cells, SULT1C2 knockdown only significantly reduced the gene expression of CS, LDH, and FACL4 enzymes (Figure 5A,B, lower panels). However, when proteins were examined using Western Blot, we consistently observed significant reductions in the protein expressions of the above enzymes in both HepG2 and Huh7 cells (Figure 6).

**FIGURE 7:**
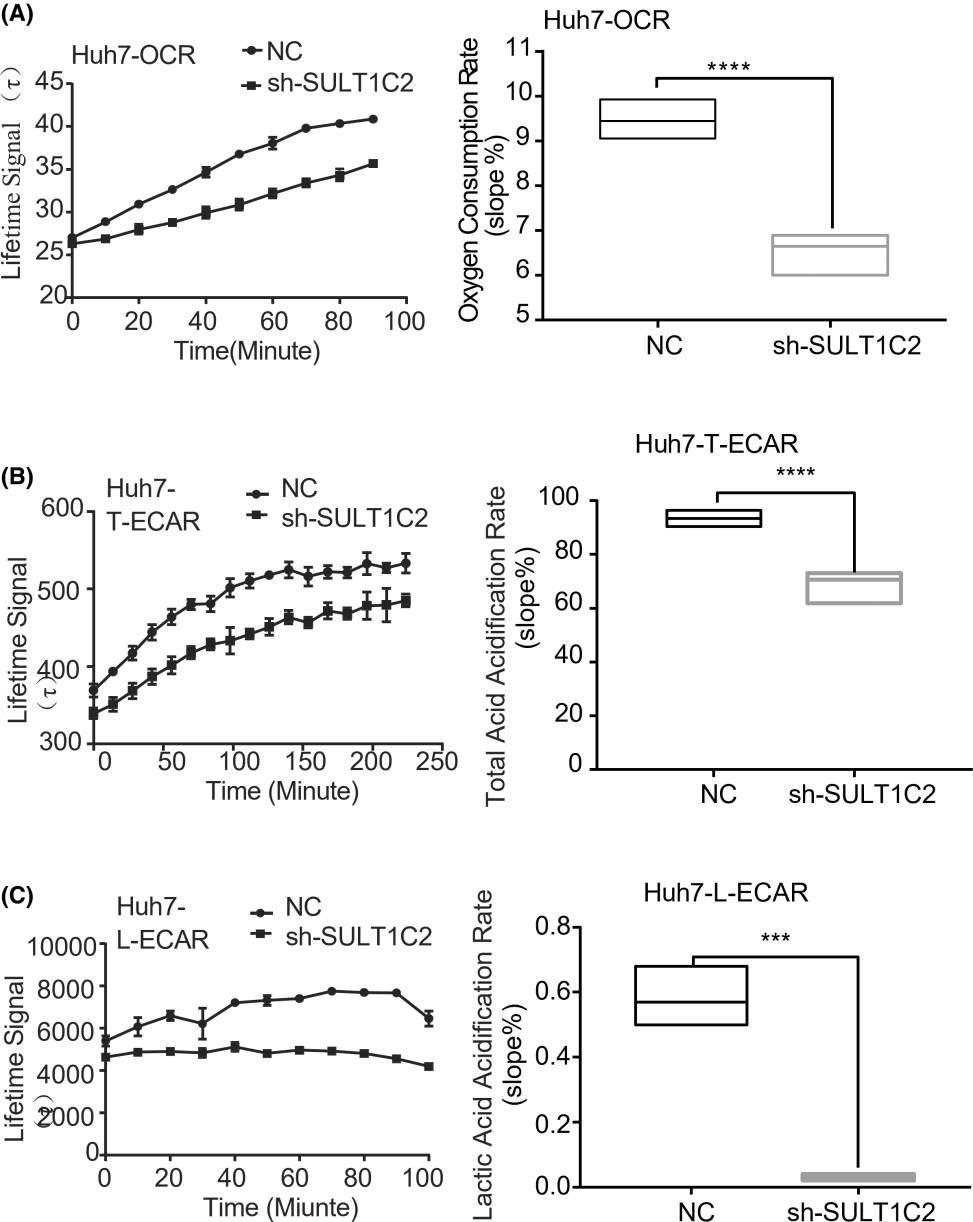
SULT1C2 knockdown significantly decreased oxygen consumption and extracellular acidification rate in Huh7 cells. (A) Data show oxygen consumption rates (OCR) in Huh7 cells with (sh) and without (NC) SULT1C2 knockdown. The change of OCR over time (left panel) and cumulative data (right panel) are shown. (B) Data show total extracellular acidification rates (T‐ECAR) in Huh7 cells with (sh) and without (NC) SULT1C2 knockdown. The change of T‐ECAR over time (left panel) and cumulative data (right panel) are shown. (C) Data show lactic extracellular acidification rates (L‐ECAR) in Huh7 cells with (sh) and without (NC) SULT1C2 knockdown. The change of L‐ECAR over time (left panel) and cumulative data (right panel) are shown. Where applicable, ***p < 0.001, ****p < 0.0001. Independent sample *t*‐test.

To determine the functional consequences of the suppressed glycolysis and fatty acid metabolism enzymes following SULT1C2 knockdown, we evaluated oxygen consumption and extracellular acidification rate in Huh7 cells. Our data demonstrated that SULT1C2 knockdown significantly decreased oxygen consumption (Figure 7A) and extracellular acidification rate (Figure 7B,C) in Huh7 cells.

### 3.7 SULT1C2 overexpression rescues the inhibitory effects of SULT1C2 knockdown in HepG2 and Huh7 cells

**FIGURE 8:**
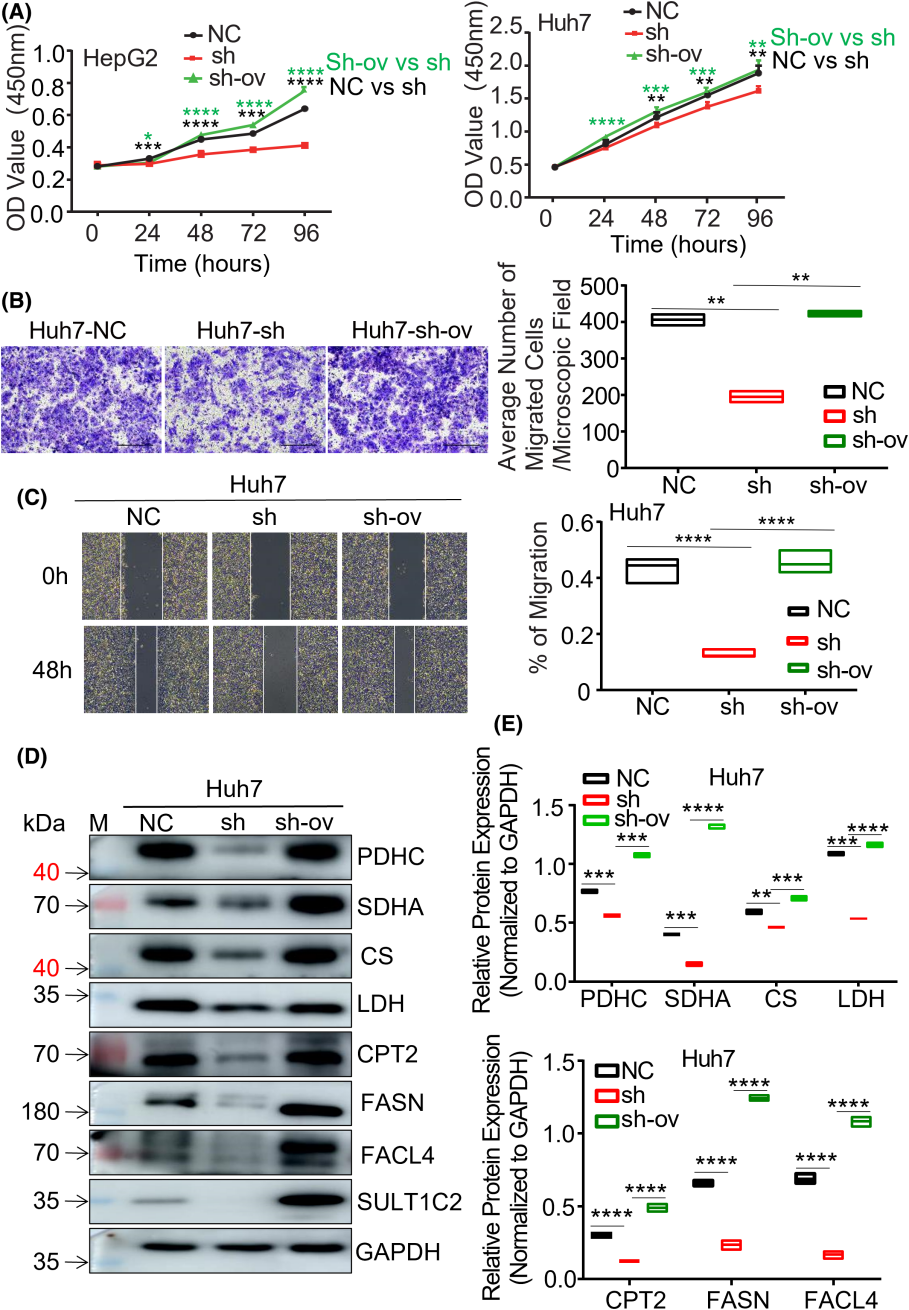
SULT1C2 overexpression rescued the inhibitory effects of SULT1C2 knockdown in HepG2 and Huh7 cells. SULT1C2‐knockdown (sh) HepG2 and Huh7 cells were overexpressed for SULT1C2 (sh‐ov) as described in Section 2. (A) The proliferation of HepG2 (left panel) and Huh7 cells (right panel) with SULT1C2 known (sh), without SULT1C2 knockdown (NC), and with SULT1C2 knockdown followed by SULT1C2 overexpression (sh‐ov) was measured using the CCK‐8 assay described in Section 2. Data show OD values at 0, 24, 48, 72, and 96 h after cell culture initiation. (B) Migrations of the NC‐, sh‐, and sh‐ov‐Huh7 cells were examined as described in Section 2. Representative images (left panel) and cumulative data (right panel) are shown. The scale bar is 200 μm. (C) Wound healing assay was performed on the NC‐, sh‐, and sh‐ov‐Huh7 cells, as described in Section 2. Representative images (left panel) and cumulative data (right panel) are shown. The scale bar is 500 μm. (D) Representative images showing the expressions of the proteins related to oxidative phosphorylation (PDHC, SDHA, CS, and LDH) and fatty acid metabolism (CPT2, FASN, and FACL4) in NC‐, sh‐, and sh‐ov‐Huh7 cells. “M” represents the visual protein Marker. (E) Quantitative data showing the expressions of the proteins necessary for oxidative phosphorylation (PDHC, SDHA, CS, and LDH; upper panel) and fatty acid metabolism (lower panel) in NC‐, sh‐, and sh‐ov‐Huh7 cells. Where applicable, *p < 0.05, **p < 0.01, ***p < 0.001, ****p < 0.0001. Independent sample *t*‐test.

Finally, we asked whether the effects of SULT1C2 knockdown could be rescued by overexpression of the same gene. Our data showed that SULT1C2 overexpression indeed reversed the effects in HepG2 and Huh7 cells that were previously knocked down for the same gene (Figure 8). The functions examined included in vitro cell growth (Figure 8A), migration (Figure 8B), wound healing (Figure 8C), as well as the expressions of the enzymes necessary for glucose and fatty acid metabolisms (Figure 8D,E).

## 4 DISCUSSION

HCC is aggressive liver cancer. Despite advanced imaging and other diagnostic measures, HCC in a significant portion of patients had reached the advanced stage at the first diagnosis. Unfortunately, there is no cure for advanced HCC. As a result, HCC is still a leading cause of cancer death. For this reason, efforts have been paid to identify novel molecules that are the HCC progression drivers and can be targeted for diagnosis, prognosis prediction, and treatment. As a result, such efforts have identified several novel molecules that are overexpressed in HCC cancerous tissues compared to adjacent normal tissues and associated with poor prognoses (e.g., ACSL4, AKR1B10, and SFN).^27^, ^28^, ^30^, ^31^, ^32^, ^33^ The clinical significance of these previously identified potential HCC progression drivers awaits further investigation.

To further understand the HCC progression, we recently performed a comprehensive screening on the molecules that might impact HCC progression using omics.^23^, ^40^ We identified SULT1C2 as a possibly novel HCC progression driver.^23^, ^40^ This current study has provided solid evidence that SULT1C2 overexpression plays a crucial role in the growth, survival, migration, and invasiveness of HCC cells (Figure 2, 3, 4). Therefore, there is no doubt that SULT1C2 is critical for HCC progression. One remaining question is the cause of SULT1C2 overexpression. Future longitudinal studies may provide insight into how SULT1C2 is involved in HCC evolution, identifying the events that drive the SULT1C2 overexpression.

We have also demonstrated that SULT1C2 overexpression led to a wide range of gene expression and metabolome changes in HCC cells (Figure S1 and S2). In addition, our data revealed that most of the SULT1C2‐associated gene expression alterations varied between hepatocellular carcinoma cell lines, underscoring the heterogeneity of this cancer. In addition, although the expression of the proteins associated with glycolysis and fatty acid metabolism was uniformly reduced following SULT1C2 knockdown, the expression of corresponding genes did not always match the protein expression (Figures 5 and 6). The data suggest that SULT1C2 regulates glycolysis and fatty acid metabolism via transcriptional and post‐transcriptional mechanisms.

Based on our discoveries, we propose that SULT1C2 promotes HCC progression via at least three mechanisms (Figure S3). Firstly, SUTL1C2 enhances anaerobic glycolysis by increasing LDH expression. Secondly, SULT1C2 augments oxidative phosphorylation by increasing the expressions of PDHC, CS, and SDHA. Thirdly, SULT1C2 elevates fatty acid metabolism by increasing the expressions of FASN, FACL4, and CPT2. It is worth mentioning that our current and previous studies also suggest the involvement of SULT1C2 in regulating other biological processes.^23^ The two prominent pathways that SULT1C2 may regulate are the spliceosome and amino acid metabolism, which warrant further investigation.^23^

The above SULT1C2‐mediated functions are via sulfation of its substrates. Although the physiological substrates of SULT1C2 remain unknown,^41^ sulfation and de‐sulfation have been shown to regulate cancer progression. For example, human sulfatase 1 (HSulf‐1) is a de‐sulfation enzyme and is downregulated in various cancers, including HCC.^42^, ^43^, ^44^ Its forced expression suppresses the growth of HCC both in vitro and in vivo.^45^ The mechanism of HSulf‐1 is believed to be the de‐sulfation of heparan sulfate glycosaminoglycans that are required for the binding of numerous growth factors to their receptors. Examples of such growth factors include fibroblast growth factor‐1 (FGF‐1),^46^ FGF‐2,^46^ vascular endothelial growth factor (VEGF),^47^ heparan‐binding epidermal growth factor‐like growth factor (HB‐EGF),^43^ interleukin‐6 (IL‐6),^48^ and interleukin‐8 (IL‐8).^49^ Hence, it is warranted to determine whether SULT1C2 works via the generation of heparan sulfate glycosaminoglycans.

The importance of our findings is the potential of the overexpressed SULT1C2 as a diagnostic marker and therapeutic target. Concerning diagnosis, human SULT1C2 is expressed in all fetal tissues except for the fetal brain.^41^, ^50^ In adults, it is mainly expressed in the stomach, kidney, and thyroid^50^ but not the liver. Although further studies are needed to screen SULT1C2 expressions under different pathological conditions, our data support that SULT1C2 overexpression can potentially be an excellent diagnostic marker for human HCC.

For therapy, the function of SULT1C2 in promoting the growth, survival, migration, and invasiveness of HCC cells suggests that SULT1C2 may be inhibited for HCC treatment (Figures 2, 3, 4). Interestingly, one study showed that the polymorphism of SULT1C2 was associated with the response to anti‐cancer drugs in prostate cancer patients.^51^ Consistent with the above findings, another study demonstrated that the glioblastoma cell line with low SULT1C2 expression but not high SULT1C2 expression responded to an anti‐cancer agent.^52^ Although the SULT1C2 expression patterns in human prostate cancer and glioblastoma require further investigations, findings from our laboratory support the development of SULT1C2 inhibitors to treat HCC.

Additionally, because SULT1C2 is selectively expressed in the adult stomach, kidney, and thyroid, SULT1C2 can be a good immunotherapy target. Hence, future studies are warranted to determine whether HCC cells can process and present SULT1C2 for recognition by the immune system.

## 5 CONCLUSIONS

This study demonstrates that SULT1C2 overexpression promotes the growth, survival, migration, and invasiveness of HCC cells. In addition, we have also shown that SULT1C2 exerts its functions by modulating gene expression, leading to metabolism reprogramming, such as augmented glycolysis and fatty acid metabolism. Hence, our data indicate that SULT1C2 is a potential diagnostic marker and therapeutic target for human HCC.

## AUTHOR CONTRIBUTIONS

**Liya Jiang:** Conceptualization (supporting); data curation (lead); formal analysis (lead); investigation (lead); methodology (lead); software (lead); validation (lead); visualization (lead); writing – original draft (supporting); writing – review and editing (supporting). **Fang Xu:** Conceptualization (supporting); data curation (equal); formal analysis (equal); funding acquisition (equal); investigation (equal); methodology (equal); software (equal); validation (equal); visualization (equal); writing – review and editing (supporting). **Chenglong Li:** Investigation (supporting); methodology (supporting). **Ting Liu:** Investigation (supporting); methodology (supporting). **Qianwei Zhao:** Investigation (supporting); methodology (supporting). **Yixian Liu:** Investigation (supporting); methodology (supporting). **Ying Zhao:** Investigation (supporting); methodology (supporting). **Yamei Li:** Investigation (supporting); methodology (supporting). **Zhengdong Zhang:** Investigation (supporting); methodology (supporting). **Xiaolei Tang:** Conceptualization (equal); formal analysis (equal); investigation (supporting); methodology (supporting); supervision (equal); validation (equal); writing – original draft (equal); writing – review and editing (lead). **Jintao Zhang:** Conceptualization (lead); formal analysis (lead); funding acquisition (lead); investigation (supporting); methodology (supporting); project administration (lead); resources (lead); supervision (lead); validation (lead); writing – original draft (lead); writing – review and editing (equal).

## FUNDING INFORMATION

This work was supported by the National Science and Technology Major Project of China (No. 2018ZX10302205); the Major Project of Science and Technology in Henan Province (No. 161100311400); the Natural Science Foundation of Henan Province (No. 222300420306); the Project of Basic Research Fund of Henan Institute of Medical and Pharmacological Sciences (No. 2022BP0103; No. 2022BP0108); the Key Scientific and Technological Project of Henan Province (No. 222102310083).

## CONFLICT OF INTEREST STATEMENT

The authors declare no conflicts of interest for this article.

## ETHICS APPROVAL AND CONSENT TO PARTICIPATE

This study was approved by the Animal Care and Use Committee at Zhengzhou University (#2020‐30).

## CONSENT FOR PUBLICATION

All authors have read and agreed to the published version of the manuscript.

## Supporting information


Figure S1–S3

## Data Availability

Data are available from the corresponding authors.
