# Supplementary material for: Sulfotransferase 1C2 promotes hepatocellular carcinoma progression by enhancing glycolysis and fatty acid metabolism
Source: Cancer Med. 2023 Mar 7;12(9):10738–54. doi: 10.1002/cam4.5759 (PMC10225225; doi:10.1002/cam4.5759)
Supplement: Supplementary file 1 — Figure S1–S3 [file CAM4-12-10738-s001.docx]

**Supplemental Materials**

**Sulfotransferase 1C2 Promotes Hepatocellular Carcinoma Progression by Enhancing Glycolysis and Fatty Acid Metabolism**

Liya Jiang^1,2☆^, Fang Xu^1☆^, Chenglong Li^3^,Ting Liu^4^, Qianwei Zhao^1^, Yixian Liu^1^, Ying Zhao^1^, Yamei Li^3^, Zhendong Zhang^4^, Xiaolei Tang*^,6,7^, Jintao Zhang*^1,5^.

^1^ Henan Institute of Medical and Pharmaceutical Sciences, Zhengzhou University, Zhengzhou,450052,Henan, China.

^2^School of life sciences, Zhengzhou University, Zhengzhou, 450001, Henan, China.

^3^School of Basic Medical Sciences, Zhengzhou University, Zhengzhou, 450001,Henan,China.

^4^BGI College & Henan Institute of Medical and Pharmaceutical Sciences, Zhengzhou University,Zhengzhou,450052,Henan, China

^5^Henan Key Laboratory of Tumor Epidemiology and State Key Laboratory of Esophageal Cancer Prevention & Treatment, Zhengzhou University, Zhengzhou, 450052, Henan,China

^6^Department of Veterinary Biomedical Sciences, College of Veterinary Medicine, Long Island University, Brookville, New York, USA.

^7^Division of Regenerative Medicine, Department of Medicine, Department of Basic Science, School of Medicine, Loma Linda University, Loma Linda, CA, USA.

☆These authors contributed equally.

*Corresponding authors:

Jintao Zhang: Henan Institute of Medical and Pharmaceutical Sciences, Zhengzhou University, Zhengzhou, Henan, China. Email: jtzhang@zzu.edu.cn.

Xiaolei Tang: Department of Veterinary Biomedical Sciences, College of Veterinary Medicine, Long Island University, Brookville, New York, USA. Email: [xiaolei.tang@liu.edu](mailto:xiaolei.tang@liu.edu).


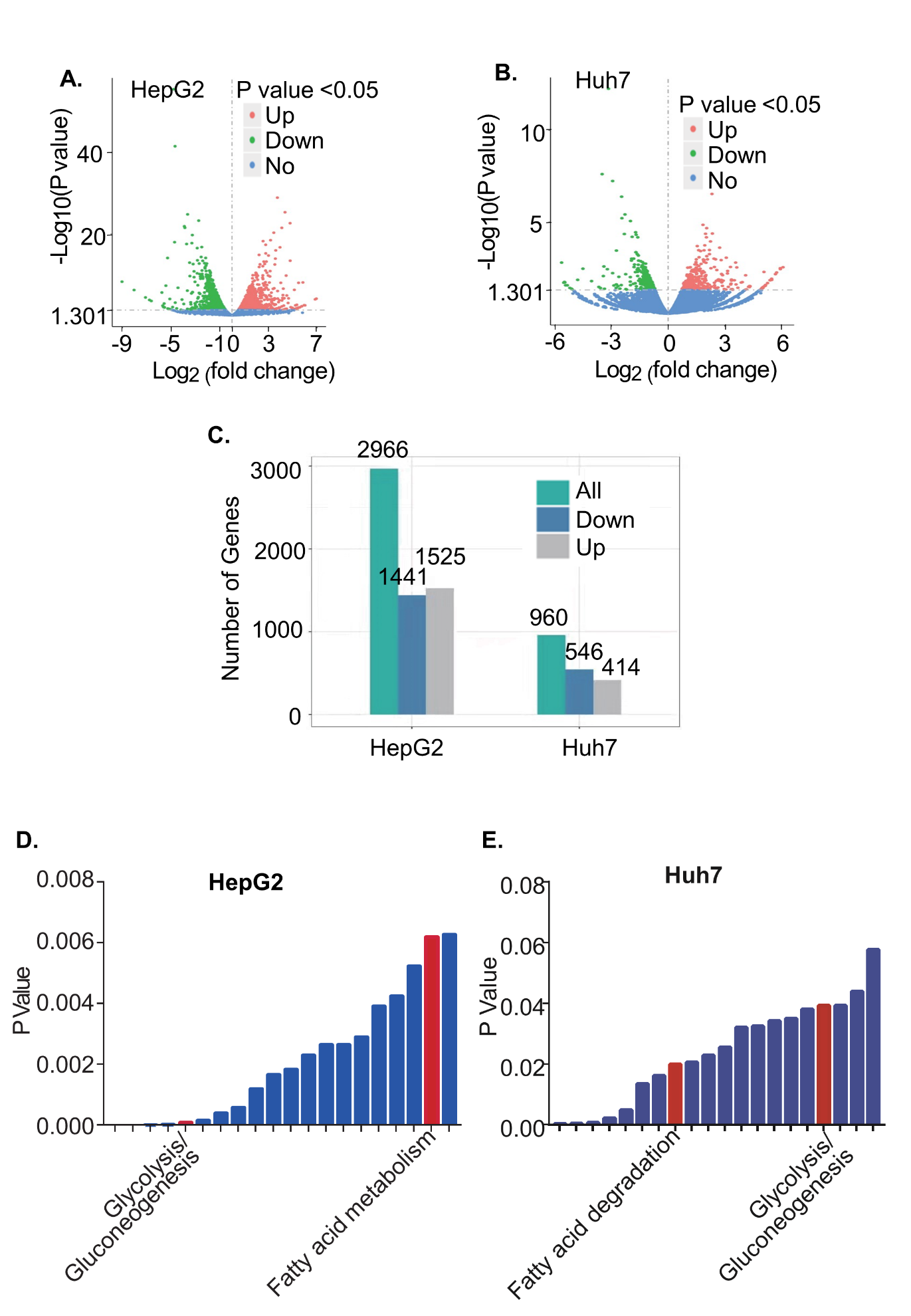


**Figure S1. SULT1C2 knockdown leads to a wide range of changes in gene expression in HCC cells. *A and B)*** Volcano plots show Log2(fold change) and –Log10(p value) of mRNA expression in HepG2 (A) and Huh7 (B) cells following SULT1C2-knockdown. Red and green dots represent significantly increased and decreased gene expression (P<0.05, horizontal dotted lines), respectively. ***C)*** Data show the numbers of the significantly changed genes in HepG2 and Huh7 cells after SULT1C2 knockdown. ***D and E)*** Data show the enrichment of significantly changed genes in various biological pathways in HepG2 (D) and Huh7 (E) cells after SULT1C2 knockdown based on Kyoto Encyclopedia of Genes and Genomes (KEGG) pathway analysis. Red bars represent the pathways enriched with the significantly changed genes shared between HepG2 and Huh7 cells after SULT1C2 knockdown.


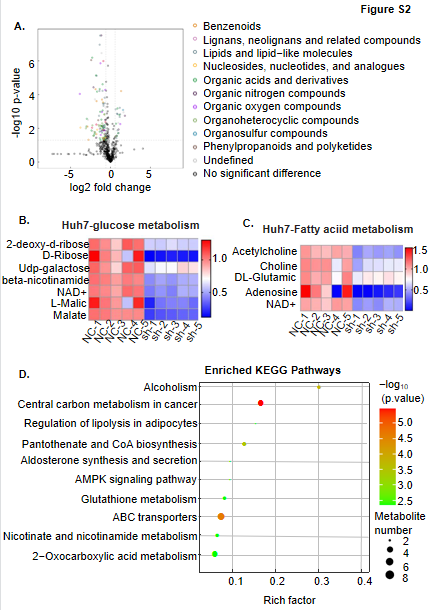


**Figure S2. SULT1C2 knockdown results in a wide range of changes in metabolome in HCC cells.** Five replicates of Huh7 cells with (sh) and without (NC) SULT1C2 knockdown were analyzed for metabolome as described in Materials and Methods. ***A)*** Volcano plot shows the identified metabolites as chemical categories. Fold change > 1.5 (or < 0.67) (vertical dotted lines) and p-value < 0.05 (horizontal dotted lines) were considered differentially expressed metabolites between groups (colored circles). Black circles represent nonsignificantly expressed metabolites. ***B)*** Heatmap shows the glucose metabolism-related metabolites that are significantly reduced after SULT1C2 knockdown. ***C)*** Heatmap shows the fatty acid metabolism-related metabolites that are significantly reduced after SULT1C2 knockdown. ***D)*** Scatter plot shows the energy metabolism-related pathways that contain significantly changed metabolites after SULT1C2 knockdown.


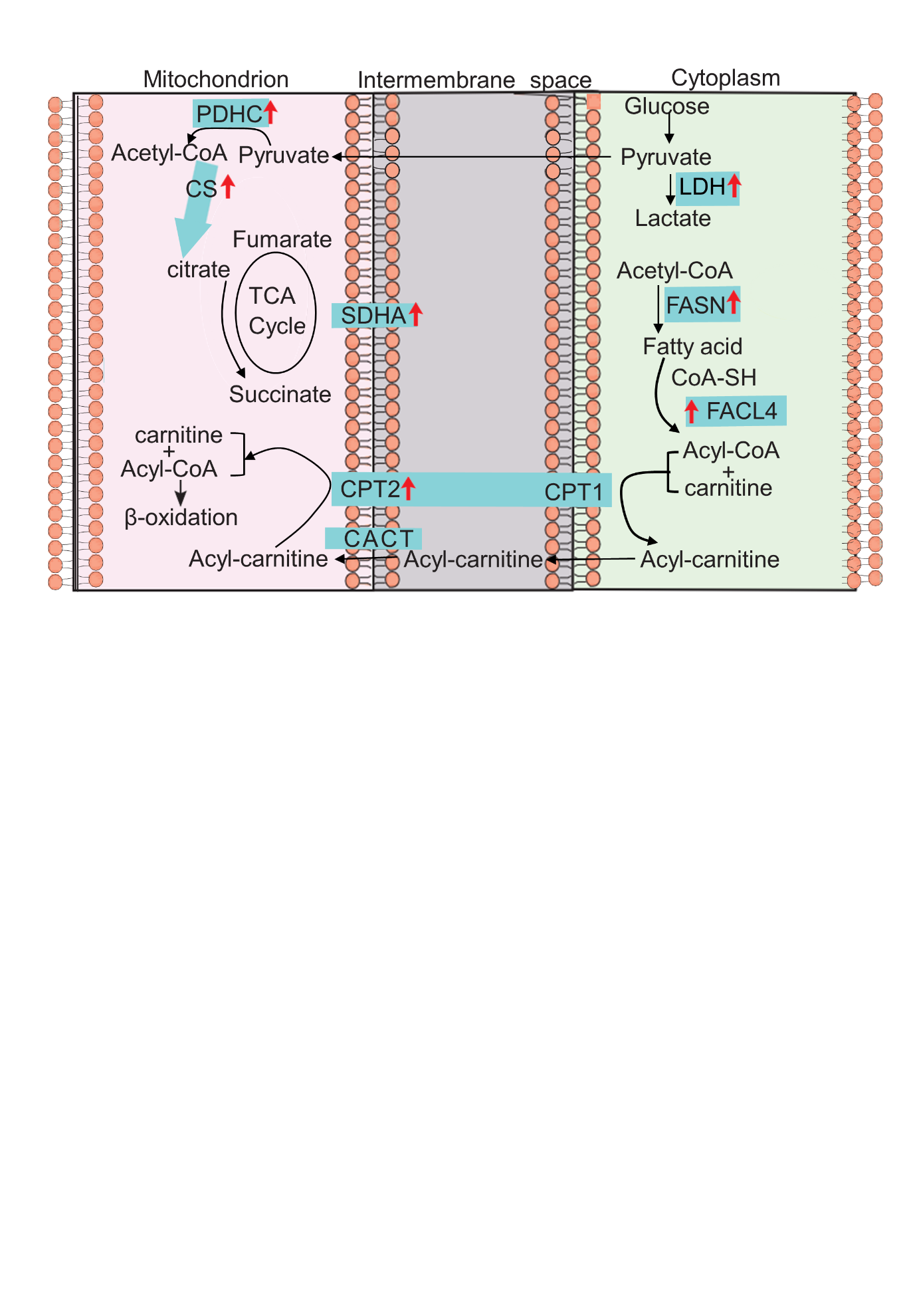


**Figure S3. A model of SULT1C2 promoting HCC progression.** SULT1C2 promotes HCC progression via at least three mechanisms. Firstly, SUTL1C2 enhances anaerobic glycolysis by upregulating lactate dehydrogenase (LDH) expression. Secondly, SULT1C2 augments oxidative phosphorylation by increasing the expressions of pyruvate dehydrogenase (PDHC), citrate synthase (CS), and succinate dehydrogenase subunit A (SDHA). Thirdly, SULT1C2 elevates fatty acid metabolism by increasing the expressions of fatty acid synthase (FASN), fatty acid co-enzyme ligase 4 (FACL4), and carnitine palmitoyltransferase 2 (CPT2).
